# Effect of best practice advisory on the administration of contraindicated medications to hospitalized patients with Parkinson’s disease and related disorders

**DOI:** 10.3389/fnagi.2023.1276761

**Published:** 2023-12-18

**Authors:** Natalia Chunga, Katherine Amodeo, Melanie Braun, Blanca Y. Valdovinos, Irene H. Richard

**Affiliations:** ^1^Department of Neurology, University of Rochester, Rochester, NY, United States; ^2^Department of Neurology, Westchester Medical Center/MidHudson Regional Hospital, Poughkeepsie, NY, United States; ^3^Department of Psychiatry, University of Rochester, Rochester, NY, United States

**Keywords:** Parkinson disease, parkinsonian disorders, hospitalization, drug contraindication, dopamine antagonists, electronic medical record

## Abstract

**Objective:**

To determine the effect of a Best Practice Advisory (BPA) on the ordering and administration of contraindicated dopamine blocking agents (DBA) to hospitalized patients with Parkinson’s disease (PD) and related disorders.

**Background:**

Patients with PD are more likely to require hospitalization and are at increased risk of complications. Administration of contraindicated DBA contributes to worsened outcomes in this patient population. Electronic medical record (EMR) warnings (also referred to as BPA) have been proposed as a way to prevent the administration of contraindicated medications.

**Methods:**

A BPA was launched in January 2020 within the University of Rochester EMR system, which alerts the provider when a contraindicated DBA is ordered in hospitalized patients with PD and related disorders. Patients with PD and related disorders hospitalized at two hospitals affiliated to the University of Rochester during a time period before (*t1*: 1/1/2019–1/1/2020) and after (*t2*: 1/8/2020–1/8/2021) the implementation of the BPA were included in this study. Epic SliderDicer was used to collect the data from the University of Rochester EMR. The number of patients who had contraindicated DBA orders and administrations in both time periods, and the number of patients who had the BPA triggered during *t2* were obtained. We compared the results before and after the implementation of the BPA.

**Results:**

306 patients with PD and related disorders were hospitalized during *t1* and 273 during *t2*. There was significantly less percentage of patients who had contraindicated DBA orders (41.5% in *t1* vs. 17.6% in *t2*) and patients who had contraindicated DBA administrations (16% in *t1* vs. 8.8% in *t2*) during *t2* (*p* < 0.05 for both comparisons). There was no significant difference between the percentage of patients who had contraindicated DBA orders in *t1* and patients with attempted orders (BPA triggered) in *t2* (*p* = 0.27).

**Conclusion:**

The results of this study increase the evidence of the potential benefit of EMR warnings for the optimization of inpatient medication management in patients with PD and related disorders. In particular, our results suggest that EMR warnings help reduce the administration of contraindicated medications, which is a known contributing factor for hospital complications in this patient population.

## 1 Introduction

Parkinson’s disease (PD) is a neurodegenerative disorder that affects over 10 million people worldwide, and the prevalence is estimated to continue increasing in the next decades (Kasten et al., 2007; Statistics|Parkinson’s Foundation, 2023). People with PD are more likely than the general population to be hospitalized. The most common reasons for admission include elective surgery (e.g., joint replacements), falls, fractures, urinary tract infections, and mental status changes (Chou et al., 2011; Gerlach et al., 2011; Low et al., 2015). Hospitalized patients with Parkinson’s disease are at increased risk of medical complications, longer stays and higher rates of morbidity and mortality. As a direct result of the hospital stay, PD patients often experience a significant decline in their level of function compared to pre-hospitalization and require a higher level of care at the time of discharge (Woodford and Walker, 2005; Gerlach et al., 2012, 2013). Several contributing factors have been identified, including adherence to complex medication regimens that require precise timing of administration, susceptibility to delirium, as well as increased risk of aspiration and falls (Aminoff et al., 2011; Low et al., 2015; Martinez-Ramirez et al., 2015). In addition, hospitalized PD patients may be administered dopamine blocking agents (DBA), which are contraindicated in this disease as they can worsen their condition and are potentially fatal in those with PD and related diseases (Lertxundi et al., 2008; Gerlach et al., 2013; Martinez-Ramirez et al., 2015).

The DBA drug class includes antiemetic medications, as well as first and second generation antipsychotics, which are commonly used in the hospital setting to treat, for example, hospital-induced delirium (e.g., haloperidol) or post-anesthesia nausea (e.g., prochlorperazine) (Golembiewski and Tokumaru, 2006; Derry et al., 2010; Schwartz et al., 2021). These medications have high affinity for the dopamine receptors, and thus, they are dangerous for PD patients who have an inherent dopamine deficiency. Serious complications can arise from the use of this drug class in PD patients, including worsening motor symptoms, mental status changes, longer hospital stays, need for a higher level of care, and potentially death (Aarsland et al., 2005; Martinez-Ramirez et al., 2015). Similarly, patients with atypical parkinsonian disorders, such as Dementia with Lewy Bodies, are also at increased risk for complications due to DBA (McKeith et al., 1992; Aarsland et al., 2005).

Considering the significant morbidity and health-related costs associated with hospitalization in people with PD and related disorders, efforts have been made to address the various contributing factors to this problem. Inpatient medication management is a potentially modifiable factor, and electronic medical record (EMR) warnings have been proposed as a potential strategy to optimize it, although they are little used (Grissinger, 2018; Lertxundi Etxebarria et al., 2021; Parkinson’s Foundation, 2023). Aslam et al. studied the effect of an EMR alert that was activated when a patient with PD or on PD medications was admitted to the hospital, and they found that patients who had contraindicated medication orders decreased after the intervention, although no significant difference in the hospitalization outcomes was found (Aslam et al., 2020). Beyond this study, there is scarce literature regarding the use of EMR warnings to prevent contraindicated medications in hospitalized PD.

Our group felt it prudent to implement an EMR warning to decrease the potential risk associated with the use of these agents in patients with PD and related disorders. An EMR warning (also referred to as a Best Practice Advisory or BPA) was created within the University of Rochester system to alert about contraindicated medications in hospitalized patients with PD and related disorders. This initiative was part the Parkinson’s Disease Hospital Optimization Project (PD-HOP). The goal of this study was to determine the effect of the BPA on the ordering and administration of contraindicated medications among patients with PD and related disorders who were admitted at two hospitals affiliated with the University of Rochester.

## 2 Materials and methods

### 2.1 Study design

This was an ecologic study aimed to evaluate the effect of a BPA on the ordering and administration of contraindicated DBA among the patients with PD and related disorders who were hospitalized at Strong Memorial Hospital and Highland Hospital, which are hospitals affiliated to the University of Rochester, located in Rochester, New York.

### 2.2 Best practice advisory

**Figure 1:**
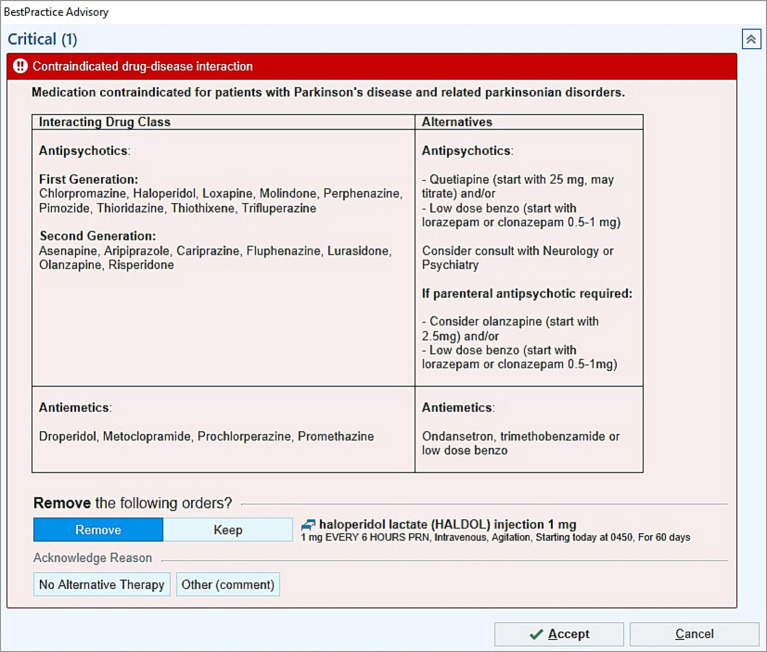
Best practice advisory (BPA) example. The BPA alerts the provider when contraindicated DBA are ordered in hospitalized patients with PD and related disorders, and it provides suggestions for alternative medications.

In collaboration with the University of Rochester Information Technology and Pharmacy Department, a BPA was created within our EMR system. The BPA was designed to alert the provider when a contraindicated DBA was ordered in hospitalized patients with PD and related disorders (Table 1 for the list of medications and diagnoses). For the BPA to be triggered, the diagnosis had to be listed in the EMR. The warning provided suggestions for alternative, safer medications including recommended dosages (Figure 1). The list of alternative medications was standard and the same in all cases; the suggestions were not tailored based on indication, as this information is not always specified in our EMR’s orders.

**Table 1:** Best practice advisory criteria.

Best practice advisory criteria	
Diagnoses (ICD-10 code)	Contraindicated DBA medications
Parkinsonism unspecified (G20) Parkinson’s disease (G20) Dementia with Lewy Body (G31.83) Multiple system Atrophy (G90.3) Progressive Supranuclear Palsy (G23.1) Corticobasal Degeneration (G31.85)	*Antiemeticsu* Droperidol Metoclopramide Prochlorperazine Promethazine *First-generation antipsychotics* Chlorpromazine Haloperidol Loxapine Molindone Perphenazine Pimozide Thioridazine Thiothixene Trifluoperazine *Second-generation antipsychotics* Asenapine Aripiprazole Cariprazine Fluphenazine Lurasidone Olanzapine Risperidone Ziprasidone

The BPA was triggered when a provider ordered contraindicated DBA in hospitalized patients with Parkinson’s disease and related disorders, according to the diagnoses and medications listed above. BPA, best practice advisory; DBA, dopamine blocking agents; ICD-10, international classification of diseases, tenth revision.

To build a list of contraindicated medications as inclusive as possible, we conducted an internet search and created a comprehensive list of all the DBA. Our team’s Movement Disorders specialists and hospital pharmacy collaborators reviewed this list to ultimately include the agents available in the USA and on formulary in our health system. Clozapine and quetiapine were not included in the list of contraindicated medications as they have predominant affinity for serotonin receptors and are less apt to worsen parkinsonism in patients with PD and related disorders (Kyle and Bronstein, 2020). In fact, quetiapine was included among suggested alternative medications.

In order to assess the functionality and determine the need of the BPA, there was a one-month period during which activation was monitored but warning was not visible to the clinicians. The warning was triggered 10 times during this one-month period, leading the University of Rochester EMR Warning Committee to approve its implementation, and the BPA was launched in the clinical setting in January 2020.

The BPA was implemented as part of the PD-HOP, a quality improvement project that also encompassed establishing a hospital-based multidisciplinary PD champion network, creating a PD educational program for hospital providers, and changing the EMR ordering system to allow custom PD medications regimens. All these initiatives were instituted in parallel.

### 2.3 Study population

For this study, we included all the patients with a diagnosis of PD and related disorders who had a hospital admission at Strong Memorial Hospital or Highland Hospital during a 1 year period before the implementation of the BPA (*t1*: 1/1/2019–1/1/2020) and after the implementation of the BPA (*t2*: 1/8/2020–1/8/2021).

### 2.4 Data analysis

Epic SlicerDicer was used to collect the data from the University of Rochester EMR. SlicerDicer is an EMR data extraction tool that provides de-identified clinical-epidemiological information on large patient populations with customizable search criteria (Saini et al., 2021). The data was obtained in February 2023.

The patient population was defined following the criteria noted above. We applied the Patients data model in SlicerDicer to obtain the cumulative number of individual patients. We used the search criteria Problem List to identify the patients with diagnosis of PD and related disorders (Table 1). For the two time periods (*t1* and *t2*), we obtained the total number of patients, the number of patients who had contraindicated DBA orders, and the number of patients who had contraindicated DBA administrations. In addition, the number of patients who had the BPA triggered in *t2* was obtained, which was used as a surrogate of the attempted orders during *t2*.

We calculated the percentage of patients who had contraindicated DBA orders and administrations in the two time periods, as well as the percentage of patients who had the BPA triggered in *t2*. Chi-square test was used to compare frequencies. A significance level of 0.05 was used. GraphPad Prism 9.0 (RRID:SCR_002798) was used for the statistical analysis.

### 2.5 Ethical considerations

This study was approved by the University of Rochester Institutional Review Board. All the data was de-identified and no personal health information was used.

## 3 Results

306 patients with PD and related disorders had a hospital admission at Strong Memorial Hospital or Highland hospital during *t1* (1/1/2019–1/1/2020), and 273 patients during *t2* (1/8/2020–1/8/2021).

During *t1*, 127 patients (41.5%) had contraindicated DBA orders, and 49 patients (16%) had contraindicated DBA administrations. During *t2*, 48 patients (17.6%) had contraindicated DBA orders, and 24 patients (8.8%) had contraindicated DBA administrations (Table 2). In addition, during *t2*, 101 patients (36.9%) had the BPA triggered.

**Table 2:** Contraindicated DBA orders and administrations before and after the implementation of BPA.

	Before BPA^a^	After BPA^b^	*p* value
Total number of patients	306 (100%)	273 (100%)	NA
Patients with contraindicated DBA orders	127 (41.5%)	48 (17.6%)	<0.0001^*^
Patients with contraindicated DBA administrations	49 (16%)	24 (8.8%)	0.009^*^

BPA, best practice advisory; DBA, dopamine blocking agents; NA, not applicable; ^(*)^ statistically significant (*p* < 0.05).

a From 1/1/2019 to 1/1/2020.

b From 1/8/2020 to 1/8/2021.

The percentage of patients who had contraindicated DBA orders was significantly less in *t2* as compared to *t1* (*χ*^2^ = 39.15, *p* < 0.0001), with an absolute reduction of 23.9%. The percentage of patients who had contraindicated DBA administrations was also significantly less in *t2* as compared to *t1* (*χ*^2^ = 6.83, *p* < 0.05), with an absolute reduction of 7.2%. There was no significant difference between the percentage of patients who had contraindicated DBA orders in *t1* and patients who had the BPA triggered in *t2* (*χ*^2^ = 1.23, *p* = 0.27), where the latter was used as a surrogate of the attempted orders during *t2*.

Additionally, among the subgroup of patients who had contraindicated DBA orders, the proportion of patients who had contraindicated administrations was 38.6% in *t1* (49 out of 127) and 50% in *t2* (24 out of 48).

## 4 Discussion

As part of the Parkinson’s Disease Hospital Optimization Project (PD-HOP), supported by a Parkinson’s Foundation Community Grant, a BPA was launched within the University of Rochester EMR in January 2020 to alert about contraindicated DBA in hospitalized patients with PD and related disorders. In this study, we found that there was a significant reduction in the percentage of patients who had contraindicated DBA orders and administrations after the implementation of the BPA.

Similar to previous studies, we found that contraindicated medications were ordered in 41.5% and administered in 16% of patients with PD and related disorders before the implementation of the BPA (Derry et al., 2010; Hou et al., 2012; Aslam et al., 2020). The high rate of contraindicated medication usage underscores the importance of implementing strategies to address this problem, especially considering that this is a potentially modifiable factor to improve outcomes in hospitalized patients with PD and related disorders.

EMR warnings have been proposed as a strategy to optimize the inpatient medication management for patients with PD and related disorders (Grissinger, 2018; Lertxundi Etxebarria et al., 2021; Parkinson’s Foundation, 2023). Aslam et al. reported that the PD patients who had contraindicated medication orders decreased from 42.5 to 17.5% after creating an EMR alert and doing in-service training sessions (Aslam et al., 2020), which is comparable to our results that revealed a reduction from 41.5 to 17.6%. These findings support the reproducibility of this type of intervention with potentially similar impact across different hospital systems. Furthermore, we found that the number of patients who had contraindicated DBA orders in *t1* was comparable to the number of patients with attempted orders in *t2* (as measured by the BPA triggers), suggesting that the orders reduction in *t2* might have been related to the BPA implementation. On the other hand, contrarily to our results, Morris et al. found no benefit from EMR alerts for preventing contraindicated orders (Morris et al., 2015). Importantly, in our study, the BPA provided suggestions for alternative medications, which could explain these divergent results and prove valuable, especially considering that the detrimental effect of DBA has been previously reported to be under-recognized among non-specialists (Esper and Factor, 2008; Chou et al., 2011).

Additionally, our study showed that not only the orders, but also patients who had contraindicated DBA administrations decreased from 16 to 8.8% after the implementation of the BPA. To our knowledge, this is the first study to show the potential benefit of a BPA on the administration of contraindicated medications in patients with PD and related disorders, which is a contributing factor for hospital complications in this patient population. Aside from the BPA, other components of PD-HOP, including the creation of a multidisciplinary PD champion network and the educational program for hospital providers, may have also contributed to the reduction in contraindicated medications.

Interestingly, the reduction of patients who had contraindicated orders was larger than the reduction of those who had administrations (absolute percentage reduction of 23.9% vs. 7.2%). In addition, among the subgroup of patients who had contraindicated DBA orders, the proportion who had administrations was less in *t1* as compared to *t2* (49 out of 127 [38.6%] vs. 24 out of 48 [50%]). These findings suggest that during *t1*, there was a higher rate of contraindicated medication orders that were ultimately not utilized. A possible explanation is that more as-needed DBA orders might have been placed during *t1*, which are not always required; at the same time, as-needed orders could have been more suitable for discontinuation or change to an alternative medication in response to the BPA during *t2*. Nevertheless, we do not have data from our hospital to support these hypotheses at this time, as we did not explore the underlying reasons for this observation. A previous study looked at the factors associated with discontinuing a contraindicated medication in patients with dopamine-requiring diseases in response to an EMR alert, and use of the medication for nausea or emesis was found to be the strongest predictor (Morris et al., 2015). Further studies would help learn more about the reasons to adopt or dismiss the suggestions of our BPA.

Considering the different impact on the reduction of orders and administrations of contraindicated medications, medication administration appears to be a better outcome measure than medication order when evaluating the effect of EMR warnings. In addition, future studies are needed to determine if the reduction in contraindicated medication administrations translates into better patient-related outcomes. Notably, Aslam et al. reported no change on the hospital outcomes after the creation of an EMR alert, despite significant reduction on contraindicated medication orders (Aslam et al., 2020); however, they did not explore the impact of the EMR alert on the administration of contraindicated medications, which could be a possible reason for their conflicting results.

It is important to note that the BPA aimed to identify patients with neurodegenerative parkinsonism. Nevertheless, other types of parkinsonism might have been captured under the diagnosis of ‘Parkinsonism unspecified (G20)’, including drug-induced parkinsonism which has been estimated to account for approximately 20% of all the parkinsonism cases (Benito-León et al., 2003). Patients with drug-induced parkinsonism might be on DBA therapy at home, which is typically continued during hospital admissions; thus, this patient group could have contributed to the patients who had contraindicated DBA orders and administrations during *t2*, despite the use of the BPA. Similarly, patients with PD and related disorders might need long-term DBA therapy, especially if they develop psychosis (Bower et al., 2018). As individual patient information was not evaluated in this study, we could not exclude those patients on long-term DBA therapy prior to the hospital admission, nor determine if the proportion of patients with drug-induced parkinsonism was similar in both time periods.

The results of this study increase the evidence of the potential benefit of EMR warnings for the optimization of inpatient medication management in patients with PD and related disorders. This study had several limitations: (1) the ecologic study design limits the extent of the conclusions, as no definitive associations or causality effects can be determined; (2) no individual patient information was analyzed, limiting the evaluation of possible confounding variables and patient-related outcomes; (3) the BPA activation and data collection were based on information documented in the EMR, which is at risk of human error; (4) the data extracted from SlicerDicer can change over time if the variables are modified in the EMR (e.g., if a patient’s diagnosis is deleted or changed, they will not be detected in subsequent searches for such diagnosis); (5) information on user experience and potential distress caused by the alert was not collected in this study. Given that the patient populations were comparable and defined by the same variables in the two time periods, we assumed that the potential effect of limitations (3) and (4) was similar in both time periods. Lastly, it is important to recognize the potential risk for EMR warning fatigue, which could lead to reduced benefit over time (Ancker et al., 2017).

In conclusion, EMR warnings are a potential reproducible strategy to improve inpatient medication management for patients with PD and related disorders. In particular, our results suggest that EMR warnings help reduce the administration of contraindicated medications. Further studies are needed to evaluate the impact of the EMR warnings on hospitalization and long-term outcomes, healthcare-related costs, sustainability over time, and combined effect with strategies for other factors leading to hospital complications in patients with PD and related disorders.

## Data availability statement

The original contributions presented in the study are included in the article/supplementary material, further inquiries can be directed to the corresponding author.

## Ethics statement

The studies involving humans were approved by University of Rochester Institutional Review Board. The studies were conducted in accordance with the local legislation and institutional requirements. Written informed consent for participation was not required from the participants or the participants’ legal guardians/next of kin in accordance with the national legislation and institutional requirements.

## Author contributions

NC: Conceptualization, Methodology, Project administration, Writing – original draft. KA: Conceptualization, Methodology, Project administration, Writing – review & editing. MB: Conceptualization, Methodology, Project administration, Writing – review & editing. BV: Conceptualization, Methodology, Project administration, Writing – review & editing. IR: Conceptualization, Funding acquisition, Methodology, Project administration, Supervision, Writing – review & editing.
